# Evaluation of complications associated with bifocal bone transport as treatment for either proximal, intermediate or distal femoral defects caused by infection: outcome analysis of 76 patients

**DOI:** 10.1186/s12891-022-05078-2

**Published:** 2022-02-09

**Authors:** Cong Peng, Kai Liu, Qi Tian, Maimaitiaili Tusunniyazi, Weiqi Kong, Haopeng Luan, Xiaokang Liu, Yan Zhao

**Affiliations:** Department of Trauma and Microreconstructive Surgery, The First Affiliated Hospital of Xinjiang Medical University, Urumqi, 830054 Xinjiang China

**Keywords:** Bone transport, Bone defects, External fixator, Infection, Complications

## Abstract

**Background:**

The purpose of this study was to evaluate the outcomes of bifocal bone transport in the treatment of femoral bone defects caused by infections.

**Methods:**

Clinical and radiographic data of patients with infected femoral nonunion treated by the bifocal bone transport at our hospital were analyzed retrospectively, from January 2008 to December 2019. Depending on the location of bone defects, the patients were divided into three groups (proximal, intermediate, and distal). The Association for the Study and Application of the Method of Ilizarov (ASAMI) criteria was applied to assess the bone and functional outcomes. Postoperative complications of three groups were documented and compared.

**Results:**

Seventy-six cases of infected femoral bone defects (31 cases of proximal, 19 cases of intermediate, and 26 cases of distal) were managed by bifocal bone transport successfully with a mean follow-up time of 30.8 months (range, 23 to 41 months). There were 58 men (76.3%) and 18 women (23.6%), with a mean age of 38.8 years (range, 23 to 60 years). The bone union was received in 76 cases with a mean of 6.9 months (range, 5 to 8 months). Pin tract infection was observed in twenty-nine cases (38.1%), 7 cases (9.2%) of muscle contractures, 3 cases (7.9%) of joint stiffness, 13 cases (17.1%) of axial deviation, 2 cases (2.6%) of delayed union, one case (1.3%) of nonunion, and none (0%) of transport gap re-fracture. One patient (1.3%) was scheduled for knee arthroplasty when bone transport treatment ended.

**Conclusions:**

Bone transport using an external rail fixator was a practical method to treat the femoral bone defects, since the satisfactory rate of bone union and limb function recovery. Complications of distal femoral bone transport were more severe than the proximal and intermedia, but the rate of complication was the least of the three groups. Soft-tissue-related complications were more likely to occur in the intermediate bone transport.

## Background

Femoral bone defects caused by the infection have still been a tricky problem for orthopedic surgeons, which is commonly brought out by open fracture and chronic osteomyelitis [1–5]. Soft tissue problems (sinus tract or necrosis) and limb-length discrepancy usually follow such bone defects, because the poor blood supply is caused by the long period of bacterial microorganisms breeding and previous multiple debridements [6–8]. Some procedures have been certified their efficacy to manage femoral defects, including the Ilizarov technique, vascularized or non-vascularized bone grafting, and the Masquelet technique [9–11]. Bone transport based on the principle of distraction osteogenesis, pioneered by Ilizarov, has gradually become the practical choice for orthopaedic surgeons to treat infected femoral nonunion. Of this technique, all possible pathologies can be resolved simultaneously, including infection, defects, length, alignment, and soft tissue problems. Via published articles [12–16], the advantages of external rail fixators are lightweight, simple intraoperative installation, convenient postoperative care, and acceptable patient compliance. However, the structure of the unilateral framework is not stable enough, which requires exquisite preoperative design and postoperative care to prevent potential complications, such as pin tract infection, axial deviation, soft tissue contracture, joint stiffness, etc.

For above, the purpose of this study was to evaluate the efficacy of bifocal bone transport in the treatment of femoral defects caused by infection, and the complications of different locations’ bone transport were compared for avoiding the potential risks.

## Methods

### Study design

After receiving the written informed consent from participants and approval from our hospital’s Ethics Committee, the medical records and radiographs were evaluated retrospectively of all patients whose infected femoral bone defect treated by single-level bone transport using a unilateral external rail fixator (Orthofix limb reconstruction system, Verona, Italy), from January 2008 to December 2019. Inclusion criteria were as follows: femoral bone defect caused by infection; sinus tract or abscess of affected limbs; positive intraoperative culture or histology supporting a deep infection; bifocal bone transport for the reconstruction of the bone defect. Patients were excluded for those younger than 18 years old, incomplete medical records, poor compliance, or follow-up time less than twenty months.

The demographic data, initial injury, previous surgical and medical treatment, comorbidities, antimicrobial utilization, biopsy, or culture results of secretions were documented. In physical examinations, the range of motion (ROM) of the hip and knee and condition of soft tissues were evaluated. The index of inflammatory was recorded, such as C-reactive protein (CRP), white blood cell (WBC), procalcitonin, and erythrocyte sedimentation rate (ESR). Cierny and Mader’s (CM) classification evaluated the degree of bone infection. The sensitive antibiotics were given to all patients intravenously for 2 weeks depending on the bacteria isolated. If no bacteria were isolated, intravenous antibiotic prophylaxis with a second-generation cephalosporin (cefuroxime 3 g, twice daily) was given at least 6 weeks before surgery and 1 week after surgery.

### Surgical technique

Patients were positioned supine on the radiolucent table, and spinal anesthesia was performed. Firstly, the affected limb’s necrotic bone and soft tissue were removed until the residual bone showed evidence of punctate cortical hemorrhage (paprika sign). The infected biopsies and cultures were collected during the surgical procedure. The surgical area was flushed with 0.9% saline under low pressure. The gloves of all participating surgeons and surgical instruments were replaced. Antibiotic-impregnated cement spacer (5 g vancomycin per 40 g gentamicin-loaded bone cement, Heraeus, Hanau, Germany) equal in length to the bone defect was then filled into the defect to receive a high level of local antibiotic concentrations. Afterward, External fixators were placed on the proximal and distal femur in an anterolateral position parallel to the respective joint. Three 4.5-mm-diameter Schanz screws were inserted at the fragment of the proximal and distal femur, and two same Schanz screws were inserted at the transport bone fragment under the guidance of the intraoperative radiography machine. Simultaneously, the desired femoral length and alignment were maintained. These screws were angled at right angles to the anatomical axis of the femur. After the external fixator sliding clamps were assembled and the external frame debugged to parallel to the axis of the femur, the minimally invasive osteotomy was performed by Gigli saw. Depending on the size of the skin loss, the direct suturing under appropriate tension, local propulsive skin flap, or vascularized free flap was utilized to cover it. Removal of the spacer was conducted when the infection was under control, which could be determined by laboratory parameters such as WBC, CRP, and ESR.

### Postoperative management

Distraction osteogenesis was conducted under the guidance of a senior orthopaedic surgeon at the 6 postoperative days, with a velocity of 0.5 mm/12 h, twice per day. Weight-bearing walking was encouraged for patients throughout the stage of distraction. The pin tract care was instructed to the patients to prevent the pin tract infection, such as washing the pin tract daily using a swab with 0.9% saline. Subsequently, radiography, WBC, ESR, and CRP were examined at 1, 3, 6, 9, 12, 18, and 24 months after bone transport. Bone grafting at the docking site was recommended to prevent docking site nonunion when the distraction stage ended.

### Data collection and outcome evaluation

Patients who met the inclusion criteria were followed by the postoperative questionnaires routinely using the smartphone after discharge. Artificially, the femur was divided into three parts equally according to the anatomical length of the femur, namely, the proximal, intermediate, and distal. The Association for the Study and Application of the Method of Ilizarov (ASAMI) criteria was applied to assess the bone and functional results. The incidence and distribution of complications in different locations’ femoral bone transport were compared.

### Statistical analysis

Data were input in a Microsoft Excel spreadsheet (Redmond, WA, USA), reported as frequencies and percentages, and then analyzed by the SPSS 20.0 software package (Chicago, IL, USA). One-way ANOVA was used to compare quantitative variables of three groups (proximal, intermediate, and distal). The chi-square test conducted comparisons of the excellent and good rate of bone and functional outcomes. Statistical significance was *p* < 0.05.

## Results

Seventy-six patients who met the criteria were included in this study between January 2008 to December 2019. There were 58 men (76.3%) and 18 women (23.6%), with a mean age of 38.8 years (range, 23–60 years). Seventy-six femoral defects (31 cases of proximal, 19 cases of intermediate, and 26 cases of distal) caused by infection were managed for bifocal bone transport using a unilateral external rail fixator, with a mean postoperative follow-up time of 30.8 months (23 to 41 months) (Table 1). The mechanisms of initial injury in all patients were summarized, including road traffic accidents (46.1%), falling injury (17.1%), direct trauma (22.3%), or other (14.4%). There were 63 cases of open fractures (82.8%) and 13 cases of closed (17.1%), with a mean of 2.8 previous operations was 3.2 (range, 1–7). The skin sinus tract took place in 43 patients, and 17 of them were in an inactive sinus scar (quiescent infection). A planned arthrodesis combined with bone transport was managed for a patient who had previous knee ankyloses. Pain and limitation of limb function were present in two-thirds of patients (knee ROM, 15–75°). With the CM classification, 51 cases in type III and 25 cases in type IV. Positive bacteria isolated was observed in 61 cases (80.2%) (Table 2). After the debridement surgery, the mean length of bone defects was 4.6 cm (range, 3.7–5.6 cm). There was no statistical significance in age (*p* = 0.105), gender (*p* = 0.647), obesity (BMI > 25, *p* = 892), smoking (*p* = 0.318), antibiotics utilization (*p* = 0.529), mode of fracture (*p* = 0.683), and duration of osteomyelitis (*p* = 0.298).

**Table 1:** Demographic of patients

Variables	Proximal (*n* = 31)	Intermediate (*n* = 19)	Distal (*n* = 26)	*P* value
Age (years)	41 (23–55)	38 (22–51)	39 (20–57)	0.105
Gender (male, female)	23M8F	16M3F	19M7F	0.647
BMI > 25(yes)	12	8	9	0.892
Smoking (yes)	18	10	16	0.318
Antibiotics (yes)	27	13	21	0.529
Mode of fracture (open)	25	17	21	0.683
Duration of post-traumatic osteomyelitis (months)	30 (22–32)	29 (22–35)	31 (16–41)	0.298

**Table 2:** Summary of patients with isolated organism and final outcome

Organism	Number of cases	Outcome
*S. aureus*	44 (57.8%)	Resolved by intravenous antibiotic therapy.
*S. epidermidis*	11 (14.4%)	Resolved by intravenous antibiotic therapy.
*E. coli*	6 (7.8%)	Resolved by oral antibiotics.
MRSA	0 (0%)	–
None isolated	15 (19.2%)	Resolved by oral antibiotics or intravenous antibiotics.

*MRSA* methicillin-resistant *Staphylococcus aureus*

**Fig. 1:**
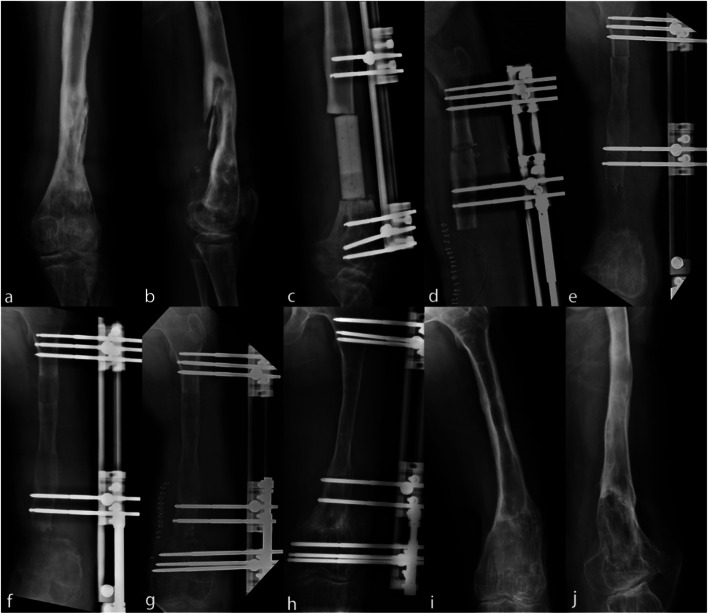
A 20-year-old male with post-traumatic osteomyelitis of the left femur was treated with single-level bone transport using external rail fixator. **a**, **b** Osteomyelitis of the left femur caused by post-traumatic infection presented by X-ray films. **c**, **d**, **e** Resection of infected bone with 7.8 cm defect, the antibiotic-impregnated cement spacer was filled and removed until infection was controlled, then treated by single-level bone transport. **f** Radiological films of 6 weeks in the distraction stage. **g**, **h** Bone transport completed with good regenerate consolidation, and docking union was achieved at four postoperative months. **i**, **j** External frame was removed with excellent bone result represented by view of X-ray at six postoperative months

Infection was eradicated in all patients, and the total bone union was received in 76 of 76 cases (100%), with a mean bone union time (BUT) of 6.9 months (range, 5–8 months). The mean external fixation time (EFT) was 11.8 months (range, 6–21 months), and the external fixation index (EFI) was 1.71 months/cm (range, 1.38–2.41 month/cm). There was statistical significance among the BUT (*p* = 0.002) and EFT (*p* < 0.001) in Table 3. According to ASAMI criteria (Table 4), the excellent and good rate of bone and functional recovery in the proximal bone transport were higher than the other (P vs I vs D, 80.6% vs 78.9% vs 69.2, 93.5% vs 89.4% vs 69.2%). The whole procedure of bifocal bone transport described in this study was shown in Fig. 1.

**Table 3:** Postoperative data of patients

Variables	Proximal (*n* = 31)	Intermediate (*n* = 19)	Distal (*n* = 26)	*P* value
Length of bone defect (cm)	4.5 (4.0–5.4)	4.8 (4.1–5.5)	4.7 (3.6–5.7)	0.610
BUT (month)	9.5 (6–13)	13.6 (7–19)	11.4 (8–15)	0.002
EFT (month)	10.8 (7–15)	14.1 (8–21)	13.6 (9–19)	< 0.001
EFI (month/cm)	2.36 (1.75–2.78)	2.31 (1.95–3.81)	2.73 (2.5–3.33)	0.733
Follow-up time (month)	31 (24–41)	30 (23–39)	29 (24–33)	0.382

*BUT* bone union time, *EFT* external fixation time, *EFI* external fixation index

**Table 4:** Outcomes of ASAMI scores in different locations

ASAMI	Location	Excellent	Good	Fair	Poor	Failure
Bone grade^a^	Proximal	4	21	6	0	–
	Intermediate	7	8	3	1	–
	Distal	5	13	6	2	–
Function grade^b^	Proximal	6	23	2	0	–
	Intermediate	7	10	1	1	–
	Distal	7	11	6	2	–

*P* proximal, *I* intermediate, *D* Distal

^a^ P vs D *p* = 0.571, I vs D p < 0.001, I vs D p < 0.001

^b^ P vs I p < 0.001, I vs D *p* = 0.643, P vs D *p* < 0.001

Pin tract infection was observed in 29 patients (38.1%) and successfully treated by dressing change combined with culture-sensitive oral antibiotics. Besides, there were 7 cases (9.2%) of muscle contractures, 3 cases (7.9%) of joint stiffness, 13 cases (17.1%) of axial deviation, 2 cases (2.6%) of delayed union, one case (1.3%) of nonunion, and none (0%) of transport gap re-fracture (Table 5). Axial deviations were corrected by adjusting the external fixator radiologically, under local anesthesia. Muscle contractures were managed by the tension-release surgery to reconstruct function. Delayed unions and nonunion were recovered by the treatment of autologous bone grafting and compaction of the regenerate intermediate connective tissue zone by the same frame (Table 6). Finally, the patient associated with knee joint stiffness obtained physical therapy to improve the ROM of the knee under the direction of a senior physiotherapist. And the knee ROM of two was recovered to 10–85° degrees. Unfortunately, the joint stiffness and intractable pain still existed in one patient, and he was scheduled for knee arthroplasty when bone transport treatment ended.

**Table 5:** Complications in the period of bone transport

Complication	Proximal (*n* = 31)	Intermediate (*n* = 19)	Distal (*n* = 26)
Pin tract infection	11	13	5
Muscle contractures	3	4	0
Joint stiffness	1	0	2
Axial deviation	4	7	2
Soft tissue incarceration	1	3	2
Delayed union	0	1	2
Nonunion	0	0	1
Transport gap re-fracture	0	0	0

**Table 6:** The treatment outcomes of complications (all patients)

Complication	Number of patients	Treatment	Outcome
Pin tract infection	29 (38.1%)	strengthen dressing change, culture-sensitive oral antibiotics or surgical intervention	Union
Muscle contracture	7 (9.2%)	surgical intervention under spinal anesthesia	Union
Knee joint stiffness	3 (7.9%)	Physical therapy	Union
Axial deviation	13 (17.1%)	surgical intervention under local anesthesia	Union
Delayed union	2 (2.6%)	surgical intervention under general anesthesia	Union
Nonunion	1 (1.3%)	surgical intervention under general anesthesia	Union

## Discussion

This study aimed to collect and assess the postoperative results and distribution of complications related to bone transport using a unilateral external rail fixator in treating femoral bone defects caused by an infection in our hospital from January 2008 to December 2019. Radical debridement combined with antibiotic spacer was necessary to ensure an infection-free limb. Bone transport using an external rail fixator was a pragmatic method to reconstruct the femoral defects and resolve the limb deformity, such as shortening, angular, sclerosis, and muscle atrophy caused by the initial injury. In this study, the rate of total bone union was 100%, and the per-patient complication was 0.82. The excellent and good rate of bone and function of the proximal group was better than the others. Pin tract infection, muscle contracture, axial deviation, and soft tissue incarceration were more likely to occur in the intermediate group, and the distal group was at high risk of joint stiffness, delayed union, or nonunion.

It’s generally recognized that the defect and deformity can be resolved simultaneously by the bone transport using an external fixator, allowing the patient to utilize the limb function earlier to prevent joint stiffness or arthritis [1–4, 17], However, this technique is also a great challenge for surgeons since the long EFT and tricky complications [18, 19]. Some interventions have been applied to shorten the EFT, such as fixation combined with an internal and external fixator, cyclic distraction and compression technique at the consolidation stage, or injection of biological agents that promote bone healing in the distraction area, etc. [7, 11, 20]. For instance, Gupta et al. [21] reported a consecutive series of 14 patients with tibia nonunion, and satisfactorily managed by bone transport using a monorail fixator combined with locking plates. All patients had a mean defect size of 6.4 cm, a mean external fixator index of 21.2 days/cm, and a per-patient complication incidence of 0.5. Furthermore, the study published by Gulabi et al. [22] presented a series of five patients with tibia nonunion successfully treated by bone transport using Ilizarov fixator combined with an intramedullary nail. The above-improved methods indeed possessed advantages in shortening the EFT, but the heavy financial burden and complex surgical procedures make them difficult to utilized widely. Hence, what’s far more important for the application of bifocal bone transport is to describe the characteristic of different locations’ bone transport and the distribution of the complications.

The high density of muscles, blood vessels, and soft tissue are distributed around the femur, especially the proximal, which facilitates bone regeneration and mineralization [1, 3, 23]. But the potential risks are kept company with these advantages. As for the surgical procedure of bone transport, the subcutaneous tissue and blood vessels are easy to be involved when inserting screws, which results in tissue necrosis and infection. In addition, the greater counterforce caused by the plentiful muscle is also left to the screws, which hinders the sliding of the transport bone segment. It has also been noticed that the distraction gap may be filled by soft tissue before the end of the distraction stage. For these, the use of a screw sleeve was recommended to plan the screws’ inserting position preoperatively and assist the insertion intraoperatively, which can reduce the probability of damaging the blood vessels and soft tissues. The periosteal formation induced by the antibiotic bone spacer after radical debridement had been suggested by the recent study, which may reduce the occurrence of soft tissue incarceration [19, 24]. And the utilization of hydroxyapatite-coated screws in the metaphysis and cortical screws in the intermediate may increase the holding force and reduce the risk of axial deviation [19, 21, 24]. Besides, the satisfactory bone union of the proximal group may be related to the selection of osteotomy line in the metaphysis of the femur, which provides fruitful blood supply for avoiding the docking site nonunion.

The autogenous bone grafting at the docking site was recommended to perform when the distraction stage finished [3, 23, 25–27]. Wan et al. [28] showed a series of 28 femoral bone defects treated by bone transport using a monolateral external fixator combined with bone grafting at the docking site, and obtained a good bone healing rate of 92.8%. Furthermore, the study published by Yin et al. [16] presented a cohort of 110 patients with bone defects of the lower extremity were treated successfully by bone transport, and the bone grafting at the docking site was applied as well. In our cohort, the bone grafting was managed at the end of the distraction stage, and satisfactory bone healing results were obtained. However, there was still one patient with nonunion and three patients with the delayed union, which occurred in intermediate and distal femoral bone transport. The delightful results were obtained with the application of bone grafting, but there was still one patient with nonunion in the intermediate group and three patients with the delayed union in the distal group. As far as we were considering, the distal site of the femur adjacent to the attachment point of the muscle resulted in poor blood supply. Fortunately, bone union was received finally after adjustment of the external fixator to compress the regenerate tissue zone until the regenerate bony parts were contacted.

Pin tract infection (38.1%) cannot be ignored, which occurred in the intermediate group and the proximal group mostly [24, 29, 30]. The reason for this phenomenon may be related to the developed musculature, rich soft tissue, and blood supply of the intermediate. This also explained why the more axial deviations and muscle contractures took place here. Previous studies have found that the occurrence of pin tract infection is related to gender, obesity, smoking, and steroid use [31, 32]. Although there is no difference in the comparison of statistical factors among the three groups in our cohort, quitting smoking and maintaining healthy living habits during treatment are conducive to the prevention of pin tract infection. Concurrently, regular X-ray examination of the affected limb was also essential to promptly prevent the axial deviation, especially applying the unilateral fixator. As for joint stiffness, the quadriceps plasty methods and their modifications may be a resolution to knee stiffness [18, 31–33]. But, complications may be brought out by the above method, including scar contracture, skin necrosis, wound dehiscence, edema, and severe pain. In our cohort, quadriceps plasty was not performed. Quadriceps plasty may be an alternation to manage knee stiffness, but the value of it is difficult to decide since the complex complications. Conversely, knee arthroplasty was a salvage approach that may be considered for the patient’s joint with poor range of motion and persistent pain.

Via published articles [11, 16, 34, 35], the application of bone transport in the treatment of infected femoral bone defects is more satisfactory than in the tibia, especially in the grade of bone healing. For instance, Sen et al. [11] showed a mean EFI of 31.8 days/cm in a cohort of 17 patients treated by the modified technique of acute shortening and re-lengthening. Additionally, Chou et al. [29] presented a mean EFI of 0.97 months/cm in a series of six infected femoral nonunions managed by the staged protocol of Ilizarov distraction osteogenesis and intramedullary nailing. In this study, however, the EFT and EFI of our cohort were higher than in the previous study. For this reason, we concluded that the intractable bone destruction was caused by the long duration of infection combined with previous multiple surgical interventions. Thus, detailed preoperative planning, skillful mastery of the external fixation framework, and methodical postoperative management should be prepared for patients with a long duration of infection. The novel combined technique described above can be a workable choice to shorten the EFT and EFI. But the risk of multiple operations’ complications cannot be underestimated, such as long incisions and extensive scars.

Last, several limitations were in this study, which was associated with its retrospective and single-center design nature. First of all, there is no mature algorithm for the resolution of complications in different bone transport locations of the femur. In addition, prospective case series with infected femoral nonunion treated by bone transport were rare. Therefore, a prospective multi-center study of large samples is crucial for clinical guidance.

## Conclusion

Pin tract infection, axial deviation remains the most common complication using unilateral external fixation. Complications of the distal site in femoral bone transport were more critical than other sites, but the rate was the least. The incidence of complications in the proximal group was comparable to the intermediate group. Soft-tissue-related complications (muscle contractures, soft tissue incarceration) were more likely to occur in the intermediate bone transport. The bone healing and functional outcomes were affected directly by these complications in the period of bone transport. It’s essential to understand the incidence and distribution of complications at femoral different locations to formulate targeted measures to deal with postoperative complications.

## Data Availability

The data sets generated and analyzed during the current study are not publicly available due to restrictions on ethical approvals involving patient data and anonymity but can be obtained from the appropriate authors as reasonably required.
